# Parents' Emotion Management for Personal Well-Being When Challenged by Their Online Work and Their Children's Online School

**DOI:** 10.3389/fpsyg.2021.751153

**Published:** 2021-10-20

**Authors:** Ramona Henter, Laura Elena Nastasa

**Affiliations:** Psychology, Education and Teacher Training Department, Faculty of Psychology and Education Sciences, Transilvania University of Brasov, Brasov, Romania

**Keywords:** well-being, emotion management, emotional intelligence, burnout, COVID-19 pandemic, online school, online work, parents

## Abstract

Parents' emotional management was highly required during the COVID-19 lockdown, as juggling as their own job moved online and with being a parent of a child whose school was online proved to be a challenge for many. Our sample was restricted to parents who had to work online from their homes while their children had to attend school online, as external imposed conditions. The present study was based on Mayer and Salovey's theory and we aimed to investigate the relationship between parents' emotional intelligence and their ability to manage their emotions during this period, hypothesizing that a higher emotional intelligence and well-developed emotional management abilities contribute to better adjustment to everyday challenges, thus contributing to keeping levels of exhaustion low. The double role played by these adults strained their resources, therefore we were also interested in their level of burnout after almost a year spent in a home turned into office and school. We also investigated the participants' level of flourishing, as described by Ed Diener, as these changes impacted differently on every parent's well-being. The analysis of the data obtained offered us the possibility of issuing a series of recommendations for parents' well-being in such a situation, as the prospect of continuing to work and learn online in future seems very real. The need to set clear boundaries between the roles played in these settings emerged as a main objective of future therapeutic interventions based on positive psychology.

## Introduction

The COVID-19 pandemic, declared as such by the World Health Organization on March 11th 2020, brought about numerous changes in everyday life. The most notable ones were those regarding work and learning, which relied on online versions. In Romania, the recommendations were to work online, where possible, after the end of the first severe lockdown, when only certain restrictions were kept. As concerning the education system, schools moved their classes online during the lockdown and during the restrictions that came with the second pandemic wave in September; from November 9th 2020 only a few students went to school (primary school students and those in their final years of studies) and only if there were no more than three cases of SARS-CoV-2 infection in their classes. Hence, most classes were online during the 2020–2021 academic year with students attending from their homes, which had already been turned into offices by their parents. If work is defined as not necessarily a paid activity, but one that is oriented by goals and directed by specific outwardly imposed tasks (Schaufeli et al., 2020), both attending school and being a parent can be seen as work. Most work activities had to be held in the same space – the family home - which may have generated friction among family members and put pressure on parents as managers of their household. Parents' well-being should have become a major concern during the COVID-19 pandemic because of the major role shifts or overlaps they encountered. However, the focus being on the medical aspects of the pandemic, each parent had to find their own strategies to face the changes it brought about.

Personal and professional success and lasting and harmonious interpersonal relationships are based on emotional intelligence (Segal, 1999), which requires a set of abilities regarding both the discrimination and monitoring of one's own emotions and others', and the achievement of goals by using such emotional information to direct thinking and behavior (Stefan and Kallay, 2010). Successful facing of challenges asks for fundamental acquisitions in cognitive, social, and decision-making domains, which are subordinated to the emotional domain, hence the controversy over the type of ability emotional intelligence belongs to: is it a cognitive or non-cognitive ability, does it involve explicit or implicit emotional knowledge, and is it an ability or outcome of a specific social and cultural context (Zeidner et al., 2001)?

Emotional intelligence can be defined as “the ability to perceive, assess, and express emotions, to access and generate feelings when they facilitate thinking, to understand emotion and emotional information, and to adjust feelings for affective and intellectual development” (Mayer and Salovey, 1997). Emotional intelligence can be regarded through a set of principles: it is a mental ability, and it is best measured as an ability. Problem solving does not always match intelligent behavior, so to measure human mental abilities the problem-solving area must be clearly stated and have well-defined subject matters. Emotional intelligence is a broad type of intelligence and it is a member of the class of broad intelligences focused on hot information processing (Mayer et al., 2016). Different perceptions of emotional intelligence can be grouped in several models: the abilities model, which considers emotional intelligence as being a cognitive ability, and the mixed models, which see emotional intelligence as a mixture of personality traits and cognitive skills (Mayer et al., 2008).

Considering emotional intelligence as based on abilities different from personality traits, four clusters can be identified: (1) *perception of emotions* – the ability to identify emotions in oneself and others, as well as in objects, art, stories, music, and other stimuli; (2) *use of emotions to facilitate thought* – the ability to generate, use, and feel emotion as necessary to communicate feelings, or use them in other cognitive processes; (3) *understanding emotions* – the ability to comprehend emotional information and the way emotions combine and progress through relationship transitions, and to appreciate such emotional meanings; and (4) *management of emotions* – the ability to be open to feelings and to regulate them in oneself and others to promote personal understanding and growth (Salovey et al., 2003). This model's premise is that emotions contain information about relationships between people, and when the relationship between two people changes, there is a change on the emotional level toward the other person (Mayer et al., 2001). These abilities help people engage in processing emotional information about their own emotions and others' and solve cognitive or behavioral problems.

Physical, emotional, and mental exhaustion can be caused by prolonged participation in emotionally demanding situations. This exhaustion demands a great amount of emotional and physical resources, as the cynicism provoked by burnout creates interpersonal distancing, while self-efficacy acts as a buffer for the burnout symptoms (Maslach, 1993; Maslach et al., 2008). Professional exhaustion is as a result of long-time stress at work, occurring when there is an imbalance between the professional area and personal abilities (Kalimo et al., 2003); it mostly affects those working with people: medical staff, social workers, therapists, teachers, educators, lawyers, or civil servants (Bakker et al., 2002). Burnout may appear along with depression, anxiety, alcoholism (Ahola et al., 2010).

Burnout can be defined as “a work-related state of exhaustion that occurs among employees, which is characterized by extreme tiredness, reduced ability to regulate cognitive and emotional processes, and mental distancing” (Schaufeli et al., 2020). These four core dimensions of burnout (exhaustion, mental distance, and emotional and cognitive impairment) are accompanied by three secondary dimensions (depressed mood, psychological distress, and psychosomatic complaints). The most obvious symptom is exhaustion or extreme tiredness and is described as a severe and serious drain of physical and mental energy, this lack of energy also impairing other capacities. Thus, burnout causes emotional impairment (reduced functional capacity to adequately regulate one's emotional processes such as anger or sadness) and cognitive impairment (reduced functional capacity to adequately regulate one's cognitive processes, such as memory or attention). Also, mental distance is present, referring to mental withdrawal and psychological detachment from the job, as an ineffective coping attempt which may increase stress at work by causing conflicts with colleagues or clients, and hence exacerbate the employee's feelings of exhaustion. The three secondary symptoms are atypical and may also appear in other physical and mental disorders, not hindering the ability to work, but are the main reason why people seek help or assistance: a depressed mood - a common reaction to disappointment or loss, distinct from mood disorder or a major depression; psychological distress - unpleasant feelings that interfere with daily activities; and psychosomatic complaints - physical symptoms that are thought to be caused, or exacerbated, by psychological factors (Schaufeli et al., 2020).

Although burnout refers to a mental state occurring in situations related to work, work is regarded from a broader, psychological perspective, including all goal-directed and mandatory tasks, thus burnout being expanded to the activity of students, sportsmen, and even parents, who are overwhelmed by being a parent, feeling emotionally distanced from their children, and ineffective as a parent (Schaufeli et al., 2020).

We consider flourishing as an indicator of high levels of well-being defined as “a pattern of positive feelings and positive functioning in life” (Diener et al., 2010). Based on humanistic and positive psychology traditions, the concept of flourishing takes into account a person's psychological and social functioning and emphasizes the presence of positive relationships, engagement, purpose and meaning, self-acceptance and self-esteem, competence, optimism, and social contribution (Diener et al., 2010). Flourishing is not a material output or a psychological state, but an emergent quality that cannot be quantified or managed directly, as it depends on contextual factors (Ehrenfeld, 2019). Considering the mental health continuum, developed by Keyes (2002), in order to describe the way individuals function, both personally and socially, people can be languishing (having low levels of emotional, psychological, and social well-being), moderately mentally healthy (being neither flourishing nor languishing), and flourishing (having high levels of emotional, psychological, and social well-being).

Flourishing refers to the state in which an individual has achieved their full potential. Although all living entities' potential is biologically set into their genome, in humans there is a second dimension to flourishing, an existential potential reflecting their striving to lead meaningful lives. Flourishing can be attained on a personal and a social level, expressing the need for both autonomy and socialization, reflected in personal wholeness, as the expression of one's uniqueness occurring in specific contexts, and in social coherence, reflecting the way an individual's actions respect the present institutional norms (Ehrenfeld, 2019).

## Methods

The main objective of our study was to investigate the relationship between parents' emotional intelligence and their ability to manage their emotions in order to adjust to everyday challenges during the COVID-19 pandemic, namely the parents working remotely at the same time as having children that were learning online. This meant they had to face numerous challenges regarding professional and personal life; therefore, we were interested to identify their level of burnout and/or flourishing. Our focus was on the Romanian society, where after March 11th, 2020, the day when WHO declared the COVID-19 pandemic, learning was mainly online for all students and the recommendation was to work online as much as possible, although a complete lockdown was not set during the 2020–2021 school year. All schools, both public and private, were obliged to comply with the safety regulations during the pandemic which stated the scenarios of online or face-to-face learning, where primary school children and pupils in the final years of studies had face-to-face classes unless there were at least three cases of coronavirus infection in their class, while all other pupils were learning online.

Our hypotheses were:

1. Emotional intelligence is positively and statistically significantly associated with flourishing.
2. Emotional intelligence is negatively and statistically significantly associated with burnout.
3. Emotional intelligence predicts low levels of burnout.
4. Emotional intelligence predicts high levels of flourishing.

### Participants

Our sample was composed of 85 parents (78 mothers and seven fathers), aged between 28 and 59 years, with a mean age of 40.35 years old. As concerning their level of studies, only 12.9% were high-school graduates, 64.7% had a university degree, and 35.3% had post-university studies. They had one child (41.2%), two children (47.1%), or three children (11.8%). The data was collected online and all participants gave their informed consent for their answers to be collected for this research and it was made clear to them that they could withdraw from the study at any moment without any inconvenience to them.

### Instruments

**Emotional Intelligence Scale – EIS** assesses emotional intelligence from an aptitude perspective. It is based on the original model proposed by Salovey and Mayer and it consists of a self-administered questionnaire with 33 items. It has a very good internal consistency, of α = 0.90 (Schutte et al., 1998) for the original scale and α = 0.93 was found for the present study, also indicating good psychometric properties.

**Test for assessing the four skills of emotional intelligence – TASEI** – comprises four parts: (a) the perception of emotions, (b) the use of emotions to facilitate thought, (c) the comprehension of emotions, and (d) the management of emotions (Caruso and Salovey, 2012). The results of this research prove a good internal consistency for understanding emotions (α = 0.80) and for managing emotions (α = 0.82) and an acceptable internal consistency for the identification of emotions (α = 0.65) and for use of emotions to facilitate thought (α = 0.63).

**The Flourishing Scale – FS** (Diener et al., 2010) was used to assess personal well-being and sense of achievement, consisting of eight items on a 6-point Likert scale ranging from 1 (Strongly disagree) to 7 (Strongly agree). As concerning its psychometric properties, it showed a good reliability in the present study (α = 0.91).

**The Burnout Assessment Tool – BAT** (Schaufeli et al., 2019) assesses the burnout syndrome as such (total score), as well as its four core components (exhaustion, mental distance, cognitive impairment, and emotional impairment) and two secondary symptoms regarding psychological and psychosomatic complaints. The authors report good internal consistencies of the BAT-C (above 0.70) and its four subscales (exhaustion: 0.92, mental distance: 0.91, cognitive impairment: 0.92, and emotional impairment: 0.90) and a Cronbach's alpha of 0.95 for the total BAT-C. Cronbach's alpha was reported at 0.90 for the composite BAT-S, whereas for psychological and psychosomatic complaints, at 0.81 and 0.85 (Schaufeli et al., 2019), respectively, similar in range to those found for the present study: the BAT-C (0.94) and its four subscales (exhaustion: 0.88, mental distance: 0.77, cognitive impairment: 0.89, and emotional impairment: 0.86) and a Cronbach's alpha of 0.95 for the total BAT-C. For the composite BAT-S, Cronbach's alpha was reported at 0.87, whereas for psychological and psychosomatic complaints, it was reported at 0.86 and 0.75.

### Procedure

We created a questionnaire on the Google forms platform by using the instruments described above and we also asked for personal data including the participant's age and number of children they had. It was distributed on social media, in groups for parents (both mothers and fathers), and the participants were also asked to further distribute the questionnaire to other parents. All answers were anonymous. We used for the analysis only those answers belonging to those who simultaneously fulfilled our two requirements set as external conditions: the adults were working online while their child/children were learning online.

## Results

The data collected was analyzed using the SPSS.23 program. We performed correlations and regression analyses to verify our hypotheses. The data analysis offered us the possibility to argue the confirmation of our hypotheses. For the first hypothesis, *emotional intelligence is positively and statistically significantly associated with flourishing*, we calculated the Pearson correlation coefficient: *r* = 0.546, *p* = 0.000. Also, flourishing is positively and statistically significantly associated with emotional abilities: with the perception and identification of emotions (*r* = 0.352, *p* = 0.001), the use of emotions for the facilitation of thinking (*r* = 0.309, *p* = 0.004), the understanding of emotions (*r* = 0.280, *p* = 0.009), and the management of emotions (*r* = 0.540, *p* = 0.000).

Parents who are able to feel, identify, and express their emotions clearly, to use them to improve their cognitive processes, to comprehend complex feelings and the way they evolve, to identify the causes of their own emotions, and to understand the relations among them as well as to manage their own emotions tend to report good well-being even if they were challenged to switch between personal and professional roles. The parents' flourishing expresses their well-being and, even more, their state of feeling comfortable in the life context they are in at the present moment, despite the pandemic constraints. Some parents may have felt being at home with their family and working at the same time as a positive.

The association of emotional intelligence with burnout was negative and statistically significant (*r* = −0.562, *p* = 0.000), underlying the fact that emotional intelligence and the ability to identify, express, understand, use, and manage one's own emotions may act as a buffer for the stressful factors that may otherwise cause burnout and thus confirming our second hypothesis, *emotional intelligence is negatively and statistically significantly associated with burnout*. Also, burnout is negatively associated with the four emotional abilities: with the perception and identification of emotions (*r* = −0.349, *p* = 0.001), the use of emotions for the facilitation of thinking (*r* = −0.324, *p* = 0.002), the understanding of emotions (*r* = −0.323, *p* = 0.003), and the management of emotions (*r* = −0.588, *p* = 0.000).

The pandemic context brought about stressful situations in both professional and personal life, emphasized by the feeling of uncertainty and possibly the fear of the consequences that infection with the Corona virus might cause. However, the parents who were aware of their own emotions, able to understand their emotions and use them in deciding and finding solutions to everyday problems, and those parents who integrate emotions in their way of thinking have a greater chance of not developing burnout syndrome, being protected by their emotional abilities (Table 1).

**Table 1:** Correlations between emotional intelligence and burnout dimensions.

		**Emotional intelligence SIE**
Core symptoms	Exhaustion BAT	−0.405^**^
	Emotional impairment BAT	−0.542^**^
	Cognitive impairment BAT	−0.448^**^
	Mental distance BAT	−0.340^**^
Secondary symptoms	Psychological distress BAT	−0.526^**^
	Psychosomatic complaints BAT	−0.499^**^

** *p < 0.001*.

Also, emotionally intelligent parents tend not to develop symptoms specific to burnout syndrome, neither core symptoms (exhaustion, emotional impairment, cognitive impairment, and mental distance), nor secondary symptoms (psychological distress and psychosomatic complaints).

Parents able to perceive, assess, and evaluate their own emotions, to access and generate emotions to facilitate thinking, to understand emotional information and its causes, and to manage their own emotions have better chances to avoid the development of the burnout syndrome. These parents have small chances of feeling extremely physically tired and emotionally drained, of presenting intense negative emotions, and feeling overwhelmed by the professional workload or relations. The emotionally intelligent parents perform cognitively and are focused on their tasks, being able to adapt to the new work conditions imposed by remote work as well as to their children's online education, which required a shift in their roles as both professionals and parents, being connected to the requirements of these changes.

Their emotional abilities helped them succeed in this pandemic context without developing difficulties in sleeping or other physical complaints, such as palpitations, stomach problems, headaches, etc., not explained by any specific physical disorder.

Not only are emotional intelligence and burnout as well as flourishing associated, but emotional intelligence can also predict a high level of flourishing and a low level of burnout. The data obtained show that 31% of the protection against burnout syndrome is explained by emotional intelligence (*R*^2^ = 0.31) as emotional intelligence has a negative contribution, predicting burnout syndrome negatively and significantly (Table 2).

**Table 2:** Regression analysis regarding the influence of emotional intelligence on burnout.

	**Coefficients**			***t***	***Sig*.**	**95.0% Confidence Interval for** ***B***	
	***B***	***Std. Error***	***Beta***			**Lower Bound**	**Upper Bound**
(Constant)	30.337	2.805		10.815	<0.001	24.757	35.916
SIE	−0.128	0.021	−0.562	−6.194	<0.001	−0.169	−0.087
Dependent Variable: Burnout							
*R* = 0.562, *R^2^* = 0.312, *F*_(1, 84)_ = 38.365, *p* < 0.001							

Our results also highlight that emotional intelligence predicts the parents' well-being, having a significant positive contribution (29%) to explaining their flourishing (Table 3).

**Table 3:** Regression analysis regarding the influence of emotional intelligence on flourishing.

	**Coefficients**			***t***	***Sig*.**	**95.0% Confidence Interval for** ***B***	
	***B***	***Std. Error***	***Beta***			**Lower Bound**	**Upper Bound**
(Constant)	13.097	4.723		2.773	0.007	3.705	22.490
SIE	0.207	0.035	0.546	5.941	<0.001	0.138	0.276
Dependent Variable: Flourishing							
*R* = 0.546, *R^2^* = 0.298, *F*_(1, 84)_ = 35.297, *p* < 0.001							

Our third and fourth hypotheses were thus confirmed, emotional intelligence being able to positively predict flourishing and negatively predict burnout syndrome.

## Discussion

The boundaries between professional and personal life were erased during the pandemic lockdown and the ensuing restrictions. In line with other studies, showing that the occurrence or fear of COVID-19 could predict negative states of mind such as stress, depression, and parental burnout, our results show that high levels of emotional intelligence can act as a buffer for these negative experiences. The highest levels of parental burnout were experienced by those who were more anxious about COVID-19 and who believed that they were at greater risk of getting this disease (Prikhidko et al., 2020).

Everyday life was completely changed by the crisis the COVID-19 pandemic created through the restrictions imposed to maintain health regulations, with a heavy toll on individuals' emotional experiences and generating burnout in parents (Le Vigouroux et al., 2021). Research showed that individuals with high levels of emotional intelligence experience fewer negative effects from work-life imbalance as compared to those who cannot manage their emotions (Bansal and Agarwal, 2017). A study on healthcare professionals' emotional intelligence and burnout showed that 20% of the variability in the level of experienced burnout is explained by emotional intelligence (Nǎstasǎ and Farcaş, 2015), a percentage lower than that we obtained for our sample of parents when the same relationship was investigated. At the same time, those who were not diagnosed with depression or burnout presented higher levels of positive emotional reactions such as hope, resilience, well-being, and flourishing (Hagan Vettera et al., 2018), underlying the possibility that the latter states could be trained and used in interventions for relieving burnout and depression.

A study comparing parental burnout before and during the lockdown found a small, but significant, difference between the two samples, with a slightly higher parental burnout score during the pandemic than for the parents interviewed 2 years earlier. The lockdown and all the other restrictions may have aggravated some of the difficulties parents were facing even before the pandemic, highlighting the weight of dispositional risk factors in the risk/resource balance of parental burnout (Le Vigouroux et al., 2021). The level of parental burnout during COVID-19 lockdown in Malaysia, for example, was between average to high, highlighting the possible threat it posed on the future mental health of those parents because of the increased emotional and mental pressure (Manja et al., 2020). The pandemic may have increased the risk of trauma, based on the lack of predictability in the known world, detachment or distancing, a lost sense of time, and a lost sense of security which, combined with the parents' worries about their economic and physical health, their concerns about their children's social isolation from peers and teachers, and the outcomes of homeschooling, may have caused the exacerbation of pre-existing mental health problems (Fontanesi et al., 2020).

Parents reported their children struggling with distance learning and they, as the proxy educators during the distance education imposed by the pandemic, experienced elevated symptoms of mental health distress (Davis et al., 2020). This new type of education, a pandemic distance learning, asks for both specific technological devices and the skills necessary to use them and, even more, teachers must use pedagogical strategies specific to online teaching (Nicolau et al., 2020). All these features meant not only teachers, but also parents, had to do a sort of home-schooling during COVID-19 pandemic for the benefit of their children. Starting a new job is a stress factor and parents during the pandemic restrictions had to face changes in their own profession which turned online and start or focus more on their teaching abilities performed with their children, all aggravating factors for burnout.

The parents' ability to perform both their professional and family roles during the COVID-19 crisis was challenged, new responsibilities being imposed on them by the new way of (tele-)working and their children's distance learning. This especially affected mothers, being the ones assuming these responsibilities (Mousavi, 2020). Mothers were at greater risk of suffering from parenting-related exhaustion in Italian culture, very similar to the Romanian one, both being of Latin origin, which still hold that women should be the primary caregiver to their children, while also managing their household and having some sort of career (Marchetti et al., 2020). Almost 25% of parents reported both anxiety and depression at clinical significance and that they had felt burned out, facts which reflect the burden and detrimental disruption of daily life routine that parents have experienced during the COVID-19 lockdown, mothers reporting more parental stress compared to their male counterparts (Johnson et al., 2020). Also, healthcare workers' burnout has become more accentuated during the COVID-19 pandemic, the professional careers of women in general, and women physicians in particular, being disproportionately affected primarily because of their caregiver responsibilities in their own families, thus underlying a situation that might also be correct for other types of professions, but not yet investigated (Padilla et al., 2021). Although a gender comparison was planned for this research, in line with other research showing higher burnout levels in mothers (Mousavi, 2020), because of the very limited number of male subjects, such an analysis could not be performed. Also, comparisons based on the parents' professions could offer interesting perspectives on the way burnout affects different types of professionals and also on which professions are more susceptible to sustaining flourishing.

In order to help face the uncertainty of the 21st century world of work, the intrapreneurial self-capital can be used, a concept seen as an individual resource comprising core self-evaluation as positive judgment of oneself in terms of self-esteem, self-efficacy, locus of control, absence of pessimism, resistance, creative self-efficacy as one's perception of one's ability to solve problems creatively, resilience, goal mastery, the ability to make decisions, and vigilance in searching for relevant information. This intrapreneurial self-capital is positively associated with eudaimonic well-being, contributing to the variance in flourishing and resilience (Di Fabio et al., 2017).

Other studies indicate that COVID-19 had indirect effects on employees' flourishing via parenting stress, based on the work–family spill over theory (Srinivasan and Sulur Nachimuthu, 2021). Emotional intelligence can be seen as a protective factor against vulnerability in times of change, implying an increased performance as well as a plausible predictor of well-being and therefore flourishing (Broli et al., 2011), results in line with our own findings. Emotional intelligence was also found as statistically significantly related to all well-being, quality of patient care, and psychological empowerment among a group of Australian aged care employees, and well-being was significantly predicted by emotional intelligence abilities (Karimi et al., 2021).

A significant interaction was identified between workplace bullying and emotional intelligence in predicting flourishing and this can be extrapolated to the crisis situation created by the lockdown, namely, emotional intelligence may lower the negative effects of workplace situations in the prediction of flourishing (Nel, 2019). Violating the work-life boundaries revealed a direct and positive relationship with burnout and a negative relationship with flourishing, emphasizing its prejudicious effect for well-being, role switching (from professional to parental) not allowing teleworkers to function optimally (Carvalho et al., 2021).

Other studies also found emotional intelligence to have a mediating role in the relationship between basic psychological needs and personal well-being, measured as flourishing and happiness (Callea et al., 2019), and also in the relationship between personal traits and positive resources predicting individual well-being (Di Fabio et al., 2018), hence the need for further analysis of this topic. When parents were able to create a flourishing state, based on their emotional intelligence and their ability to use it in everyday situations, their burnout levels were low. Therefore, we can suggest intervention programs designed to develop parents' emotional intelligence as a protective factor for the occurrence of burnout and also as incentives for developing parents' well-being and flourishing.

Recent research highlighted the importance of implementing support groups for parents of children with special needs (Nǎstasǎ et al., 2018), taking into account that these parents were not professional caregivers or teachers – a situation similar to what happened during the pandemic distance education when parents had to turn to their skills to perform the teacher's role for their own children. We suggest that support groups focused on developing emotional abilities and on the most effective emotional management strategies to cope with the pandemic situation, to avoid burnout, and even more, to thrive during it, achieving a high flourishing state, could be helpful in such unpredicted and strenuous contexts as living during the pandemic restrictions. Our results highly emphasize the need for parents to participate in training for the development of their emotional intelligence abilities, as factors influencing the coping mechanisms employed in facing adversities such as those generated by the COVID-19 pandemic, could be done in specific contexts – training like that descried by Nǎstasǎ et al. (2021) and Nǎstasǎ and Fǎrcaş (2012). Also, school counselors could promote the development of emotional intelligence abilities for the adults (parents and teachers) involved in raising and educating children, with our study as a strong argument in favor of the benefits of a high emotional intelligence and highly developed emotional intelligence management abilities.

Future therapeutic interventions within the positive psychology paradigm, such as the development of the intrapreneurial capital (Di Fabio et al., 2017), could be the foundation of increasing the parents' levels of eudaimonic well-being. Learning how to manage their own lives in terms of long-term and priority goals while keeping in mind gratitude for what they already have may be a shift in perspective for parents struggling with the multitude of tasks generated by strenuous contexts, such as the pandemic, but also for future situations which cannot even be imagined at the present moment. Emotional intelligence could mediate the relationship between adverse situations and burnout or flourishing. More subjects' characteristics should be investigated in future research. Also, the emotional self-regulation strategies used by parents could be discovered in a psychological intervention focused on the development of emotional intelligence, but especially on learning strategies to co-regulate emotions in the couple to improve parenting abilities. When one of the parents allows themselves to depend on the partner in the couple, they create a base of security and a source of power and resilience, which gives them the freedom to explore opportunities in the environment, to take risks, and to develop autonomously (Johnson, 2020). If parents learn to recognize their attachment needs, communicate them coherently to their partner, and accept responsibility for their own emotions (followed by clear identification and correct labeling of their own emotions, their discrimination, and processing), they have a good chance to gain safety, satisfaction with life, well-being, and flourishing. At the same time, the quality of family relationships is an important factor in terms of the physical, mental, and emotional health of its members (Johnson, 2008).

Our participants were mainly women as in Romania it is usually the mothers who take care of children and are concerned with their education and they were more available to share the difficulties they encountered during the pandemic; this lack of gender equal representation could be considered the main limitation of our study. The questionnaire was created using the Google forms platform and was distributed in groups for parents on social media. We found that mainly women answered, although there were also men in those groups, this being an aspect that could be addressed in future research on the direction of men's involvement in raising children or their readiness to answer questions about fatherhood. Our focus was on the impact of the pandemic on adults' coping strategies; therefore, we collected answers from those with a parental status in order to identify the compound impact of the demands of online schooling for their children and working online in their own profession. Also, it would be an interesting future study to identify their children's emotional intelligence abilities as it could mediate the relationship between the adults' emotional intelligence abilities and their level of burnout respectively flourishing as the children's abilities could impact the parents' well-being. We also consider the fact that we did not compute the differences between the parents' levels of burnout/flourishing based on the number of children they had a limitation. This will be part of a future investigation, focused mainly on the differences in parents' well-being according to their number of children.

The major contribution of our study is the investigation of the relationship between emotional intelligence and well-being at its two opposite poles, burnout and flourishing, during the COVID-19 pandemic context. We could not identify any study or article in the main databases researched (Web of Science Core Collection, Scopus, and Science Direct) where all three variables (emotional intelligence, burnout, and flourishing) were analyzed together; therefore, we may say we opened a new line of research.

## Data Availability Statement

The original contributions presented in the study are included in the article/supplementary material, further inquiries can be directed to the corresponding author.

## Ethics Statement

Ethical review and approval was not required for the study on human participants in accordance with the local legislation and institutional requirements. The patients/participants provided their written informed consent to participate in this study.

## Author Contributions

The authors equally contributed to this article, and both read and agreed to the published version of the manuscript.

## Conflict of Interest

The authors declare that the research was conducted in the absence of any commercial or financial relationships that could be construed as a potential conflict of interest.

## Publisher's Note

All claims expressed in this article are solely those of the authors and do not necessarily represent those of their affiliated organizations, or those of the publisher, the editors and the reviewers. Any product that may be evaluated in this article, or claim that may be made by its manufacturer, is not guaranteed or endorsed by the publisher.
